# Motivations for the Use of Video Game Streaming Platforms: The Moderating Effect of Sex, Age and Self-Perception of Level as a Player

**DOI:** 10.3390/ijerph17197019

**Published:** 2020-09-25

**Authors:** Luis Javier Cabeza-Ramírez, Sandra M. Sánchez-Cañizares, Fernando J. Fuentes-García

**Affiliations:** Faculty of Law, Business and Economic Sciences, University of Córdoba, Puerta Nueva s/n, 14071 Córdoba, Spain; sandra.sanchez@uco.es (S.M.S.-C.); fernando.fuentes@uco.es (F.J.F.-G.)

**Keywords:** motivation, new media, streaming, Twitch, uses and gratifications, media usage, video streaming service, video game

## Abstract

A particularly striking new phenomenon in recent years is the live streaming of video games through popular platforms, such as Twitch. This study focuses on the motivations and types of use underlying viewer participation in live streaming platforms. Based on the uses and gratifications theory, this paper aims to analyse how three basic motivations are related to the use of video game streaming platforms. Furthermore, it examines the moderating effects that significant variables, such as the audience member’s age, sex or self-perception of level as a player may exert on this relationship. The results reveal that the three types of motivations are positively associated with use of the platform, although notable differences appear, with informational motivations outweighing entertainment and social motivations. At the same time, no moderating effects on the results of the proposed model were found for the heterogeneity stemming from sex and age. Conversely, the influence of informational motivations on the use of these platforms is moderated by the self-perception of level as a player.

## 1. Introduction

Crowdsourced streaming services allow almost anyone to generate and broadcast content over the internet [1]. This is a phenomenon that is becoming ever more prominent, especially in the field of gaming. These days, millions of users opt to spend some of their leisure time watching content-creators engage in their favourite hobby on platforms such as Twitch [2]; some users even prefer doing this to actually playing video games [3]. In addition, the rise of e-sports has been revolutionary for professional gamers and fans [4]; these mass events have been streamed primarily on Twitch, but also on other platforms that have been emerging in light of the former’s success (Mixer, Facebook Gaming, Caffeine or YouTube Gaming). These services do have a connection to standard broadcast media such as television; however, they represent emergent formulas in which the participative interaction between streamer and viewer challenges the traditional concepts established in audience studies [5].

In 2019, more than 359 million hours of content were made available on Twitch alone, with 11.79 billion hours watched and a monthly average of 1,200,000 concurrent viewers [6]. The popularity of such streaming services has skyrocketed, representing an emerging new form of consumption. Moreover, the lockdowns imposed in response to the Covid-19 crisis have doubled the average audience, with the platform experiencing unprecedented growth in the streaming of content in all languages [7]. Video game streaming is comprised of three essential components: the live public playing of the game for a potential audience; varying levels of interaction between streamer and viewer; and the coexistence with the video inside and outside of the streaming [8]. In today’s gaming culture streaming is hugely influential. In this context, it can be described as the process of real-time transmission of video content on video games through a live broadcast platform, such as Twitch, Mixer, Caffeine, Facebook or YouTube Gaming. According to Horban et al. [9], the phenomenon encompasses both the people involved in playing the game and producing the content, as well as the individuals outside of this process who act as spectators and interact with the streamer or with other viewers.

In recent years, there has been an emergence of interesting new studies exploring the burgeoning phenomenon of the live streaming of video games. According to Burroughs and Rama [10] “Twitch is an ideal space to study audience adoption and participation within streaming sites, but also the industry’s re-articulation of the video game space that solidifies streaming as a dominant mode of spectatorship, participation, and play”. In this respect, Taylor [11] points out how the content generated on these platforms and the endlessly expanding audience is altering the way we currently understand communication media. Other studies focus on new content creators (streamers), addressing the context in which video games and streaming become professionalized, and examine the contribution made to shaping contemporary digital culture [12]; the difficulties streamers experience when their audience grows [13] and how it can affect their performance as players [14]; as well as the different ways in which they stream and create content with their viewers [15]. Among the lines of research focused on the audience are those that explore the motivations and types of use underlying viewer participation [10,11]. However, little is known about how motivations vary in specific cultural contexts or about the influence that certain moderating variables can exert. This study seeks to fill that gap by analysing the predictive power of three basic motivations for use and whether the inclusion of moderating variables affects the results. Based on the widely-applied theoretical framework of the uses and gratifications (U&G) theory [12], we propose a simple psychological model with three motivations (tension release/entertainment, social and informational) and three possible moderating effects (age, sex and self-perception of the level as a player). The empirical results of this analysis allow comparisons with other contexts and previous studies [2,16,17]. They can also contribute to a better understanding of user behaviour on this type of platform as well as helping to improve the design of more effective communication strategies.

## 2. Literature Review

Research on live streaming platforms is still relatively scarce [18]. A significant proportion of the studies carried out have focused on the audience. In said field, one of the most notable lines of research consists of analyses of viewers’ behaviour and degree of commitment to live streaming [16,19,20], mainly through the study of the audience’s motivations [2,17]. These types of studies draw on different theoretical frameworks, such as the U&G theory [2,17], social identity theory [20], or on multidisciplinary approaches based on cultural studies of gaming or media [16,19]. These works point to common patterns of consumption [19]. They show how, compared to other media, viewers’ motivations for participating in live entertainment seem to have a stronger social and community basis [17], although they report different degrees of importance of the various motivations for live streaming [21,22].

### 2.1. The Uses and Gratifications Theory

One of the most established theoretical approaches for understanding the motivations for and uses of media consumption is the U&G theory; this framework is particularly appropriate for interpreting the early stages of new forms of mass media [23]. The theoretical approach is based on five basic precepts: (1) the use of communication media is motivated by certain goals, and said behaviour is functional and has consequences for people; (2) the use of communication media is a way of satisfying desires or interests, such as seeking information, solving personal dilemmas or reducing uncertainty; (3) social and psychological factors mediate communication behaviour; (4) media compete with other forms of communication to be chosen, to capture attention and to attract use; and (5) people tend to be more influential than media in person–media relationships [24].

The U&G approach is a psychological communication perspective that explains the effects of media in terms of purposes, functions or uses (U&G); it focuses on the choice patterns of receivers as active users [24]. Despite its limitations [25], the U&G framework emphasizes the role of the active audience and has become a natural candidate for shedding light on new online media [26]. Choi et al. [27] summarize this approach, highlighting how the audience tries to meet certain psychological needs when choosing a medium and how the gratifications sought are the ones that motivate use. Kircaburun et al. [28] note how, according to this perspective, audiences are motivated by two types of gratification: gratifications sought, which refer to users’ expectations; and gratifications obtained, which are the needs fulfilled by the use of the media. Both affect the choice, frequency and intensity of media use [24].

The U&G theoretical approach is becoming more prominent with the rise of new interactive media in which users enjoy a greater degree of participation [29]. Models developed from U&G theory have evolved and are being widely used and tested in a range of contexts, for example, to explore the use of social networks and their effects [28,30], the use of video games [31], virtual social worlds [32], smart speakers [33] or innovations in the way of watching television [34,35]. Live streaming platforms have come to represent a novel hybrid of conventional television, YouTube and a social network; the content they provide is primarily—though not exclusively—video games. Consequently, they involve at least three basic motivations: tension release/entertainment, social and informational. Based on these, associations have been established between the type of streaming and the video game genre played, affective motivations, tension release, search for information, learning and personal and social gratifications obtained [36]. In addition, some of the models developed link motivation and use [2], or motivation and degree of live-stream viewer engagement [17].

Some recent studies applying the U&G theory have found that the use of recommendation systems, a lack of self-control and self-esteem, along with the motivation of searching for information lead to excessive use of this type of service [37]. Conversely, others point to affective gratification and identification as the most influential factors in users’ experiences of these platforms [38]. However, further empirical evidence is needed for a more in-depth understanding of user behaviour. In their intercultural analysis, Oh et al. [39] found significant differences between Eastern and Western culture in various linguistic and psychological dimensions. In this regard, there have been relatively few studies that account for the possible impact on user behaviour of variables that may be a source of heterogeneity in the sample. Such variables may expand, mitigate, cancel out or invert the proposed associations between motivation and use. They include users’ sex or age [27,40] or even other variables more closely related to users’ profile as gamers, such as their self-perception of their level as a player or their experience [41].

### 2.2. Motivation and Use in Video Game Streaming Platforms

In the 1970s, Katz, et al. [42] developed 35 needs related to the media and classified them into five categories: cognitive (acquisition of knowledge, information, understanding); affective (emotion, pleasure, feelings); personal integrative (credibility, status, stability); social integrative (interactions with family, friends, streamers and other users); and tension release needs (escape, entertainment). In the 1980s, McQuail [43] grouped needs into four reasons for media use: information, self-identity, social integration and interaction, and entertainment. As Khan [29] indicates, the U&G perspective has been refined over the years to offer a better understanding of users’ motivations, pointing out that the classifications can vary widely. In this regard, Dholakia, et al. [44] highlighted six categories in their model of consumption and participation in virtual communities: obtaining information, giving information, building a reputation, developing relationships, recreation and self-discovery. In line with the proposed objective and previous research in the field of streaming platforms [2,17], this study focuses on three of the main motivations that can help predict the use of this type of platform: tension release-entertainment, obtaining information, and social motivations linked to communication and a sense of belonging.

#### 2.2.1. Entertainment and Tension Release Motivation

The most recreational aspects of the use of platforms like Twitch—such as simple entertainment and tension release—have previously been identified as significant motivations for using live video game streaming services [16,19]. Users access these platforms in search of entertainment, seeing them as an alternative to social networks or television. Here, they can watch others play without feeling the tension that actually playing the game itself may at times entail—they can relax and simply enjoy watching others play [14]. In this regard, previous studies of social networks have found that entertainment is the strongest predictor of the use of Facebook, Instagram, Twitter and Snapchat [45]. In the same vein, in their analysis of live streaming channels, Hsu, et al. [46] found that the gratifications obtained in terms of entertainment and sociability boost users’ loyalty to the different channels.

Another aspect related to entertainment is the tension release or escapism that users can achieve by going on such platforms. Watching streams of video games relieves stress, distracts the audience from their daily activities and partly takes them out of their real lives; that is, it also fulfils a psychological need for escapism [18]. This construct is controversial in the literature on video games: it can have positive connotations, being associated with an adaptive use for stress control; and also negative, linked to a wide range of psychological problems or addictive uses [47]. The present study takes the positive aspect, focusing on the entertainment motivation. Previous studies report results indicating that visual appeal and escapism are important factors that affect and precede enjoyment [48], and consider escapism an appropriate predictor of consumption behaviour [49].

Therefore, we propose the following hypothesis:

**Hypothesis 1** **(H1).** The stronger the actual/potential users’ tension release/entertainment motivation, the greater their use of video game streaming platforms.

As noted in the methodology section the sample of users is made up of both current users of this type of platform and individuals who, given their interest in video games as a hobby, have the potential to be users in the future. Tension release and entertainment have been considered together as a single variable.

#### 2.2.2. Social Motivation

Chat rooms play an important role in video game streaming platforms, fostering sociability between regular viewers, moderators and streamers, who welcome and interact with users live [17]. Social motivations—such as communicating with other viewers, making new friends or being part of a community—attract users who, in addition to enjoying their favourite video game, socialize with each other and/or with the streamer [50]. Gros, Wanner, Hackenholt, Zawadzki and Knautz [16] point out that another name for this type of platform is Social Live Streaming Services (SLSSs), emphasizing this aspect. Furthermore, Hu, Zhang and Wang [20] show how group identification is reinforced by the co-experience consisting of participation, cognitive communion and resonant contagion. Hilvert-Bruce, Neill, Sjoblom and Hamari [17] reveal how communities are formed in places like Twitch, where viewers use chat rooms to talk, laugh and joke about the content they view.

Numerous previous studies have shown how social motivations impact media use or the consumption of video games [46,51,52]. As Li, Wang and Liu [18] indicate, the audience on these platforms has important social demands, including connections between streamer and viewer, or among audience members. Wulf et al. [53] note that intention to watch is influenced by the relationship between audience and streamer, while Hu, Zhang and Wang [20] find that viewers’ identification with audience groups is positively associated with intention to continue using the platform. Therefore, hypothesis 2 can be formulated as follows:

**Hypothesis 2** **(H2).** The stronger the actual/potential users’ social motivation, the greater their use of video game streaming platforms.

#### 2.2.3. Informational Motivation

Cognitive motivations relate to the need for the acquisition of information, understanding and knowledge [54]. Therefore, platforms such as Twitch help the audience to learn strategies, develop gaming skills and acquire information [18]. Video game streaming services have become an important source of information. Johnson and Woodcock [55] show their impact on the entire video game ecosystem and industry, revealing how they help build links between video game developers and streamers. Streaming platforms influence players’ expectations regarding new releases, with users going to these platforms as an audience member to find out information, to see how new releases are tested live or how older titles are updated, or to watch indie game releases.

The search for information has been positively associated with the hours users spend on streaming services, as well as the number of streams they choose to watch [2]. Platforms like Twitch are in constant evolution as sources of information that complement traditional information systems. They even offer an alternative to internet search engines [22]. Users can turn to this type of service to stay up-to-date on their favourite video games, learn new game strategies or follow tournaments and events [19]. Therefore, hypothesis 3 is proposed as follows:

**Hypothesis 3** **(H3).** The stronger the actual/potential users’ informational motivation, the greater their use of video game streaming platforms.

#### 2.2.4. Heterogeneity and the Moderating Effects of Sociodemographic Variables

Video games are no longer seen simply as a teenage hobby; they have become a common, meaningful form of adult leisure activity that is even overcoming persistent gender barriers [56]. Today’s society has broken away from the established themes surrounding video games. Pioneering research, such as that of Griffiths, et al. [57], was some of the first to provide evidence of a sociodemographic gamer profile far removed from the stereotype of socially-withdrawn young people or adolescents, revealing instead a varied segmentation with emerging minorities in terms of gender, broader age groups, educational level or income. Data from Newzoo [58], the consulting firm specializing in video games, corroborate this evolution first identified almost 20 years ago. They show how the current profile of the video game consumer has shifted: gamers are now predominantly adults, with only a small gender gap, mostly full-time workers with middle/high income levels and living in a family unit. However, as Long and Tefertiller [59] point out, even though the gaps in sex and age groups are shrinking, these types of variables still have a notable effect. In their analysis of video streaming platforms, the aforementioned authors find that men are more likely to watch and prefer live video game streaming, suggesting that gender could moderate the motivations that lead women and men to use these types of services. Consequently, the following hypothesis can be proposed:

**Hypothesis 4** **(H4).** Heterogeneity in terms of gender affects motivations for using video game streaming platforms.

Furthermore, along with gender, the link between age and the consumption of video games remains a controversial aspect [60]. Previous research suggests that as age increases, there may be notable differences in preferences, motivations for gaming and even styles and identification as a player [61]. As such, motivations for using video game streaming platforms could be expected to similarly vary. That said, the phenomenon of video game streaming platforms is relatively recent, so there could be an age gap in favour of younger users, which in turn could determine differences in their motivation. Therefore, the following hypothesis is proposed:

**Hypothesis 5** **(H5).** Heterogeneity in terms of age affects motivations for using video game streaming platforms.

**Figure 1:**
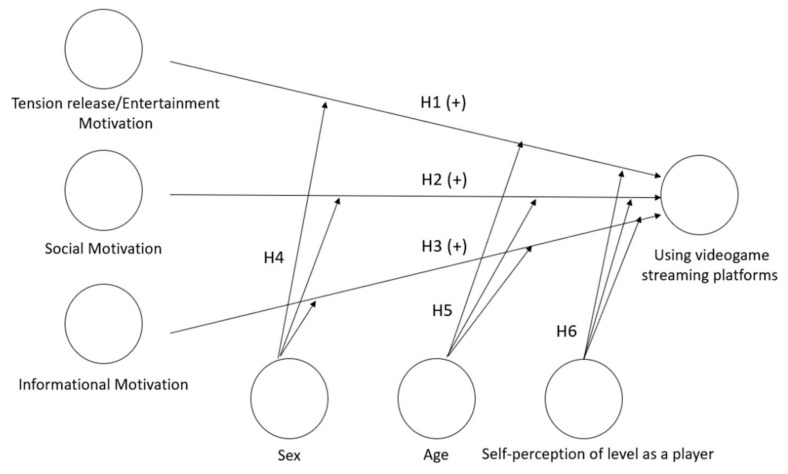
Research model.

In addition to these two variables, there may be others that are determinants of the use of platforms such as Twitch. In this regard, the player’s experience or skill level has rarely been taken into account in analyses of motivation. However, studies such as those by Manero, et al. [62] in educational video game contexts show how age and gender do not exert an influence in terms of increasing interest in particular topics, but the level of the player or his/her game preferences does have an effect in terms of increased interest and effectiveness. In the same vein, Orvis et al. [63], state that video game experience significantly predicts affective and motivational learning outcomes, including the motivations of entertainment, satisfaction or time spent participating in instructional games. Therefore, self-perception of level as a player is established as a variable that could affect the motivations for using and use of video game streaming platforms, since it is expected, in line with previous studies such as those of Gandolfi [19], that advanced users’ motivations may differ from those of users who are less skilled at video games. Consequently, the last hypothesis of the model summarized in Figure 1 is raised:

**Hypothesis 6** **(H6).** Heterogeneity in terms of self-perception of level as a player affects motivations for using video game streaming platforms.

#### 2.2.5. The Use of Video Game Streaming Platforms

Sjoblom and Hamari [2], Hilvert-Bruce, Neill, Sjoblom and Hamari [17] and Gandolfi [19] have highlighted how the motivation to watch others playing is instrumentalized in use: through hours spent viewing, following, supporting or contacting streamers and following specific games. Figure 1 summarizes the research model based on three of the main constructs of the U&G approach; these constructs are represented as latent variables and group the types of uses mentioned above.

## 3. Methodology

### 3.1. Sample

The target population is Spanish users of live video game streaming services. A total of 1050 valid responses were obtained from a survey to collect primary data conducted on Surveymonkey between September and November 2019.

Before posting the final survey, a pre-test was performed on a control group (N = 12) in order to correct any phrasing or inconsistencies. The link to the survey was published on a range of platforms, social networks and video game forums, as well as in various universities and educational centres. Although the total number of questionnaire responses was slightly higher, incomplete ones were removed from the sample, yielding the final database. To obtain a diversity of usage habits, the selection criteria for the survey respondents were not restrictive, meaning there is a high degree of randomness.

Table 1 shows some sociodemographic data of the sample. Male respondents can be seen to predominate, with the mean age reflecting the youth of the respondents, although the sample includes individuals of up to 74 years of age. The educational level is significantly high, with more than 85% of respondents having completed upper secondary or higher education. Finally, it is noteworthy that students represent almost half of the sample, although there is also a high percentage of full-time workers.

In addition, respondents stated that they mainly use their smartphone as a gaming platform, followed by the PC. As for the channels on which users watched streamers playing video games, Twitch was the main one, followed by YouTube Gaming, far above Mixer or Caffeine.

A total of 11.5% of respondents stated that they are or have been streamers and most (73.2% of the sample) do not exceed 10 h a week of gaming. Only 7.2% spend money on Twitch or other viewing platforms, mainly through subscriptions. However, more than 30% consider themselves to be an expert or pro gamer, compared to 36% who see themselves as novices or amateurs and one-third of the sample who rated themselves as regular.

Regarding the preferred video game genres, action/adventure video games stands out, followed by real-time strategy and sports; genres related to FPS (6.8%), survival horror (8.4%) or Japanese RPG (8.5%) were rarely chosen as favourites. A genre classification based on Vargas-Iglesias [64] was used, with the addition of some specific genres such as Japanese RPG.

### 3.2. Measurement Instrument

The questionnaire design was based on previous research papers [2,16,19,65], adapted in order to gain an understanding of the determinants of the use of these platforms.

The questionnaire was divided into four blocks:

(a) Sociodemographic characteristics
(b) Game attributes, preferred platforms for playing/viewing, time spent weekly and self-perception of level as a player.
(c) Motivations for playing/viewing content.
(d) Uses of the platform.

Each of the analysed constructs in the proposed model was measured through a series of items, with the source of the scale specified in Table 2. Thus, the Tension release/entertainment motivation variable is measured as in Gros et al. [16] and Andreassen, Billieux, Griffiths, Kuss, Demetrovics and Mazzoni [65], through three items related to the need for entertainment, the use of this type of viewing platform as an alternative to other networks or TV, or users playing/watching video game streaming to forget about their problems. The variable for social motivations is also measured with three items based on the scales used by Gros, Wanner, Hackenholt, Zawadzki and Knautz [16] and Gandolfi [19] about communicating with other users, making new friends or being part of a community. Three items from the scale by Gros, Wanner, Hackenholt, Zawadzki and Knautz [16] have also been used for measuring informational motivation; they refer to learning new strategies, staying up-to-date on video games, or following tournaments.

Lastly, five indicators have been used for the endogenous variable “Use” following the work of Gros, Wanner, Hackenholt, Zawadzki and Knautz [16], Gandolfi [19] and Sjoblom and Hamari [2].

All items have been measured on a 5-point Likert scale, ranging from 1 (not important) to 5 (very important), except for viewing hours, which is divided into five intervals of increasing numbers of hours, as in the study by Sjoblom and Hamari [2].

### 3.3. Procedure Validity and Reliability

The model and hypotheses presented have been tested using the Partial Least Squares (PLS) procedure. The measurement model is validated by assessing the reliability of the individual indicators through their loadings (Table 2), as well as the internal consistency and convergent and discriminant validity (Table 3).

Most of the outer loadings exceed the cut-off value of 0.707 suggested by Carmines and Zeller [66], with the exception of some items related to social motivations, tension release and uses. However, since they are in different constructs and do not fall below the value of 0.4 established by Hair, et al. [67] as the cut-off for the removal of an indicator, they are left in the model as they can help extract useful information available in the indicator to generate a better latent variable score and, equally, the rest of the measurement indicators for the constructs that verify the discriminant validity (Table 3). In addition, the bootstrapping procedure performed confirms the validity of the loadings through the Student-t test, with all *p*-values being <0.01.

Regarding internal consistency and convergent validity, Table 3 presents the reliability and validity measures assessed through Cronbach’s Alpha, composite reliability and average variance extracted (AVE) for each of the three motivation constructs and the dependent variable on main uses. The first two measures surpass the value of 0.7 suggested by Nunnally and Bernstein [68] as an appropriate level for acceptable reliability. With regard to convergent validity, all the constructs satisfy the AVE criterion proposed by Fornell and Larcker [69], exceeding the cut-off of 0.5; that is, each construct explains at least 50% of the variance of the assigned indicators.

Table 3 also indicates that the discriminant validity requirement is fulfilled, as the square root of the variance shared between the construct and its measures (AVE), shown in the values on the main diagonal (in bold) exceed the correlations between each construct and any other (the rest of the matrix).

Following the analysis of the main model, the moderating effect of the three factors under analysis (sex, age and player level) was studied through a multigroup analysis, PLS-MGA, together with the permutation test [70].

## 4. Results

**Figure 2:**
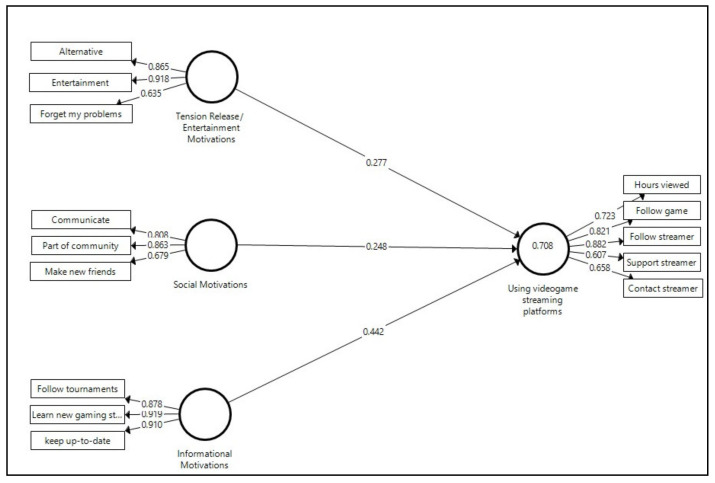
Estimated structural model. p < 0.01 for all the parameters; SRMR = 0.099.

The estimated structural model (Figure 2) shows how the three motivation constructs explain 70.8% of the variance in terms of the level of use of video game viewing platforms, such that the proposed model presents a fairly high goodness of fit. According to Chin [71], an R2 value above 0.67 can be considered substantial. The standardized root mean square residual (SRMR) is below 0.10, indicating the goodness of fit of the model [72].

Moreover, after confirming the absence of multicollinearity (VIF < 5 for all indicators), the three path coefficients—positive and significant according to the bootstrapping procedure (Table 4)—indicate that the first three hypotheses proposed are accepted. It can thus be affirmed that stronger tension release/entertainment motivations, as well as social motivations and informational motivations, generate a higher level of use of video game platforms. However, the value of the coefficient for the relationship between informational motivations and use is higher (0.442) than the other two coefficients, which are more similar in weight. Consequently, the type of motivation related to the need to stay up-to-date, follow tournaments and learn new strategies is the one that has the greatest influence in terms of higher levels of use of video game streaming platforms. The two other types of motivation—tension release/entertainment and social—also have a positive and statistically significant influence on use, albeit weaker, with the former showing slightly greater weight in the relationship with the endogenous variable.

After analysing the model for the complete sample, we test how the sociodemographic variables sex and age as well as the self-perception of level as a player affect the influence of the respondents’ motivations on their level of main uses. To that end, the bootstrap-based multi-group analysis PLS-MGA was carried out for each of these variables to analyse the differences in the coefficients depending on the categories of each of these possible moderating variables.

When applying and interpreting the PLS-MGA, it is first necessary to check that the requirement of measurement invariance is met in order to be able to make an appropriate comparison between the groups in terms of the estimated standardized path coefficients in the structural relationships of the composites. To that end, the procedure to assess the measurement invariance of composite models (MICOM) developed by Henseler, et al. [73] is applied. It is a three-step procedure involving the following elements: (1) compliance with configural invariance; (2) compliance with compositional invariance; (3) equality of means and variances. If the requirements are met in the first two steps it indicates partial measurement invariance, while if the third step is also verified it indicates total measurement invariance.

After applying MICOM for the three factors whose moderating effect is to be analysed, it was confirmed that the compositional invariance requirement is not met in the case of sex and age so there would be no point in interpreting the results of the multigroup analysis for these two variables. In other words, neither the sex nor the age of the respondent can be considered to have a moderating effect on the relationship between the different types of motivation and the level of use. These two factors are not a source of heterogeneity in the results of the structural model estimated for the complete sample. Therefore, hypotheses 4 and 5 are not accepted.

Following Gros, Wanner, Hackenholt, Zawadzki and Knautz [16], the self-perception of level as a player was measured on a five-point scale (novice–amateur–regular–expert–pro). However, when applying MICOM and PLS-MGA, the variable must be dichotomous in order to be able to analyse the differences between the two groups or categories of the variable. Thus, the self-perceived level was recodified as: (a) basic player, which combines the categories novice, amateur and regular; (b) advanced player, which combines the categories expert and pro. Table 5 shows the results of the MICOM analysis for this factor in each of the constructs of the structural model. In all cases, compositional invariance was confirmed but not compliance with the third step of equality of means and variances. Therefore, partial measurement invariance is established and the groups can be compared.

Table 6 shows the results of the MGA using two alternative approaches, Henseler’s MGA [72] and the Welch–Satterthwait test. Both procedures yield the same result, indicating that only self-perception of level as a player gives rise to significant differences in the relationship between informational motivations and the level of use. The path coefficient for this relationship is significantly higher for low-level players, while the two alternative motivation groups (tension release/recreational and social) show a higher value for advanced players, although the difference from the other type of player does not allow a statistical interpretation. Based on these results, we can accept hypothesis 6.

## 5. Discussion and Conclusions

One of the most promising lines of research on the live streaming of videogames is focused on user behaviour. The first review of the relationship between the use of these types of services and various psychological variables [18] suggests the need for more empirical evidence on this issue. In this context, the results of the present study provide several theoretical and practical contributions.

### 5.1. Theoretical Implications

Examining the results obtained in more depth, it can be seen that informational motivations are found to be the strongest positive predictor of the use of streaming platforms. This finding is in line with those obtained by [2,16,17,36] among others, and those reported in previous studies on the use of social networks [74,75]. The surprising thing about this result is that this construct is the strongest determinant of use. It has been found to have similar importance in a specific analysis of YouTube, where the values for informational motivations tend to outweigh others [29]. However, the consumption of video content on YouTube differs markedly from live streaming. As Haridakis and Hanson [76] point out, YouTube videos are watched in search of information and shared for entertainment and social interaction; that is, they correspond to different behaviour, as the audience can search for and select specific content. In contrast, in a live stream the watching occurs concurrently to broadcasting and is thus somewhat unpredictable [53]. It is worth noting that not all studies on live streaming have found that cognitive gratifications obtained have high predictive power; for example, Long and Tefertiller [59] claimed that the search for information was a relatively weak motivation for watching live streams in China. This contradiction suggests that the context and other variables may be influencing the results. According to Gros et al. [16], there are different types of specific broadcasts on streaming platforms, which can facilitate informational motivations; for example, informal streams with more flexible structures enable interaction with streamers in relaxed environments, where it is easier to ask questions about the game or strategies. Indeed, previous studies, such as that by Zimmer, et al. [77], have shown the importance of information for users of this type of service. They highlight five dimensions of information behaviour in users (information service quality, information user, information acceptance, information environment and time). In addition, results of other studies indicate that enjoyment and the information dimension affect the amount of content generated by users, although the intention to continue contributing to the platform is primarily influenced by the social capital of the streamer [78]. However, not all aspects related to the type of use aimed at meeting information needs are positive; it has also been found that they could be determinants of excessive use of such platforms [37].

At the same time, the search for information is also aimed at learning about video games as a product. Indeed, Gros, Wanner, Hackenholt, Zawadzki and Knautz [16] report a strong association between the action genre and informational motivations. This type of video game tends to be a costly AAA release (AAA means high economic risk in the video game industry); thus, users may visit these platforms to weigh up their possible purchase by viewing the development of the game beforehand. Therefore, high values for informational motivations may suggest a need to account for the context and incorporate moderating variables when developing motivational models. Other variables could include the type of channel watched on the platform or the genre of the video games considered [79], and even the way in which information is interacted with and transmitted [80]. Genre studies has been a major element of game studies for years. Some video games even revolutionize their genre and take it to another level [81]. Furthermore, there are genres associated with certain cultures or particular preferences [82,83]. In this regard, the results obtained here could be explained by the obvious difference between Chinese culture and Spanish culture [59]. Furthermore, 45% of the respondents indicated a preference for action games and approximately 70% of the sample reported that their gaming level was not particularly high, which would be linked to increased informational needs.

With regard to the moderating variables taken into account, it was found that neither sex nor age had any effect. This result could indicate that—as is the case with videogames in general—live streaming is moving away from sociodemographic stereotypes that could influence motivation and use of these platforms [56,57]. In the field of research on live streaming or videogames, the genre of the game has often been used as a control variable [79,84]. However, it is less common to include variables related to self-perceived characteristics as a player. In this case, a higher self-perceived level as a player strengthens the influence of the three analysed variables on use. This finding is in line with the concept of effectance developed by White [85], according to which the motivational system is driven by competence and progress toward the acquisition of skills and knowledge [86]. This opens the door to analyses accounting for both the actual and self-perceived characteristics as a player in motivational studies focused on live streaming and videogames [87].

On the other hand, the model presented here yielded very similar values for the other two motivations studied, albeit clearly lower than the informational values. Both (tension release/entertainment and social) were positively associated with the use of the platform. These results were partly expected as they support previous research on live streaming [18]. Tension release satisfies the audience’s need for escape. Users turn to such platforms as a way to relieve stress, escape from their everyday lives and forget about their problems by watching others engage in one of their favourite hobbies. As Hamari and Sjoblom [4] indicate, the degree of escapism correlates with the frequency of watching live video. Watching video games distracts and entertains the audience, especially in informal or even competitive environments [2]. Previous analyses have shown how entertainment is the most basic motivation for using such platforms. Entertainment tends to be the main motivation in the sphere of live streaming [16,17,59], social networks, such as Facebook, Twitter, Instagram and Snapchat [45] and YouTube [29]. However, we did not find it to be the case in this study, which is in line with previous behavioural studies on the use of traditional media showing that the entertainment motivation and intention to use are weakly related but that informational motivations are more closely linked to intention [88]. This finding is consistent with the U&G approach applied and the existence of more ritualized and instrumental media orientations [24]. As such, analyses of Twitch users in different contexts would help shed light on this aspect. In addition, it has previously been shown that learning about video games is a significant reason for starting to watch live streams [2]; in the sample studied, approximately 41% stated they only watched between 0 and 3 h a week, which may correspond to early stages of use of the platform.

In terms of social motivations, they were expected to have greater predictive power. However, several contributions to date point to the need to optimize the social function of streaming. Ref. [2] claim as much, highlighting the need to increase the sense of belonging and community for the different audiences. In this regard, although these platforms are considered innovative and full of possibilities, as the number of viewers increases, communication becomes more and more unidirectional [20], interactivity is lost and they increasingly come to resemble a conventional medium such as television. Along these lines, [89] explains how the social dimension is affected by the size of the different channels. Mass streams mean that more people use the chat room and their activity becomes more disordered, ruling out possibilities for real interaction. Furthermore, over time, communities become increasingly closed off, with their own sets of rules. This fact was confirmed during the research, since certain channels stipulate requirements for using the social interface tools; for example, users must follow the streamer; stay on the channel for a certain length time, which can vary between 10 min and an hour, before being able to send a message; go through moderation filters; and observe certain rules of behaviour. Therefore, the results regarding social motivation can be understood in view of the effect exerted by the time the user spends on the platform; that is, new or relatively inexperienced users could use these platforms more for informational motivations or for entertainment [89], while more established, experienced users would assign more importance to social functions.

### 5.2. Practical Implications

The theoretical contributions reveal different practical implications, both for video game developers as well as for those running streaming platforms and streamers or professional gamers who are engaged in streaming. Differences among the three basic motivations in relation to the use of the platforms indicate, at least in the Spanish context, that informational motivations are the main predictor. This finding underscores the importance of contextual or cultural differences when it comes to developing effective marketing strategies; that is, Twitch and other platforms could take advantage of this knowledge about user motivation when selecting the first channels that the viewers see, or in the categories recommended by the platform according to the country or IP of origin.

Another key issue arising from the empirical results is that it cannot be affirmed that either sex or age has a moderating effect on the main motivations leading to use. One might expect, for example, that social motivations would be more important for a younger audience, or for one or other of the sexes; however, the analyses carried out indicated that this heterogeneity had no such effects. This result has serious implications for the management of live streaming channels since, in principle, it means it is not necessary to segment audiences based on these variables as there are no observable differences in their motivations. Instead, targeting audiences using variables relating to player profiles would be much more appropriate; for example, streamers could pose a question before users access their channel, or immediately afterwards, to focus their streamed content. The versatility of this type of platform enables the implementation of tools that make it possible to constantly adapt the stream to meet the audience’s needs, which would markedly improve the effectiveness of the broadcast.

Lastly, the fact that social motivations ranked third as a predictor of use is relevant since, as discussed in the previous section, the socialization of the audience could affect their loyalty and degree of commitment. New users or those with low gaming skills may feel excluded; this points to the need for tools to be implemented that facilitate the socialization of new viewers. In this regard, [90] propose designs for gamification mechanisms to foster interaction between streamers and audiences, and among users.

### 5.3. Limitations and Future Lines of Research

This study makes several theoretical contributions to the literature and opens up potential avenues for future research on the live streaming of video games. Nevertheless, the study is subject to several limitations and further analysis is required to help validate the findings. First, the sample was drawn exclusively from Spanish audiences, so future studies should collect data from other countries. This will provide valuable information for analysing the influence of cultural differences on the audience’s motivations and use. Second, a convenience sample was collected on social networks, video game forums and Spanish Twitch channels, so it may not be representative of the entire population. Third, the study focuses on three basic motivations and the influence of a number of moderating variables. However, the results obtained and the complexity of this new medium of mass communication call for more complex psychological models to expand on the moderating effects of new variables related to gamer profiles, or user profiles on Twitch or other platforms (genre played, games followed, type of channel used, etc.). Finally, in the limited research on live streaming, most of the analyses have been configured as static studies, capturing the different motivations at a given point in time. However, these motivations can evolve as a user improves their skills or as their preferred genre for playing/watching changes; as such, we believe there is a need for longitudinal studies that capture the evolution of the user on the platform and enable experimental designs that determine cause–effect relationships.

## Figures and Tables

**Table 1:** Sociodemographic profile of the sample and profile of use of video game platforms.

Variable (Unit)	Categories	Value	Variable (Unit)	Categories	Value
Gender %	Male	69.4%	Weekly hours spent watching streaming %	0–3 h	70.0%
	Female	30.6%		3–7 h	14.1%
Age (years)	Mean	26.88		More than 7 h	15.9%
Education %	Primary level	3.6%	Weekly hours spent gaming %	0–3 h	40.4%
	Lower Secondary level	9.2%		3–7 h	19.4%
	Upper Secondary Level	55.3%		More than 7 h	40.2%
	University	21.9%	Self-perception of level as a player %	Novice	26.2%
	Master/PhD	10.0%		Amateur	9.7%
Employment/Current activity %	Student	48.1%		Regular	33.7%
	Full-time employee	29.6%		Expert	23.4%
	Part-time employee	4.4%		Pro	7.2%
	Student and employee	13.0%	Favourite video game genre % *	Action/Adventure	45.0%
	Unemployed	4.9%		Real-time strategy	23.2%
				Adventure	20.4%
				Sports	20.3%
				Online FPS	19.2%

Note: * More than one option could be selected (percentage exceeds 100%).

**Table 2:** Measurement model. Descriptive statistics and loadings.

Item	Source of Scale	Loading	Mean (SD)
MOTIVATION 1. Tension release/Entertainment			
For entertainment	[16]	0.918	2.45 (1.464)
Alternative to social networks/TV	[16]	0.865	2.25 (1.387)
I play/watch to forget my problems	[65]	0.635	2.28 (1.318)
MOTIVATION 2. Social			
To communicate with other viewers	[16]	0.808	1.45 (0.873)
To make new friends	[19]	0.679	1.32 (0.750)
To be part of the community	[19]	0.863	1.48 (0.918)
MOTIVATION 3. Informational			
To learn new gaming strategies	[16]	0.919	2.25 (1.351)
To keep up-to-date with my favourite video games	[16]	0.910	2.22 (1.376)
To follow tournaments and events	[16]	0.878	2.05 (1.349)
USE			
Hours viewed 1-(0-2); 2-(3-5); 3-(6-10); 4-(11-20); 5- (>20)	[2]	0.723	1.63 (1.188)
To follow a specific streamer	[19]	0.882	2.16 (1.417)
To follow a specific game	[19]	0.821	2.26 (1.424)
To support a streamer	[16]	0.607	1.23 (0.600)
To contact a streamer	[16]	0.658	1.23 (0.615)

**Table 3:** Internal consistency, convergent and discriminant validity of the measurement model.

	Cronbach’s Alpha	Composite Reliability	AVE	Fornell–Larcker Criterion			
				Inform	TenR/Ent	Soc	Uses
Informational	0.886	0.929	0.814	0.902			
Tension release/entertainment	0.744	0.853	0.665	0.763	0.815		
Social	0.727	0.829	0.619	0.539	0.512	0.787	
Main uses	0.797	0.860	0.555	0.706	0.741	0.628	0.745

**Table 4:** Path coefficients and t-value (structural model).

Hypothesis	Expected Sign	Path	t-Value
H1: Tens rel. * → USE	+	0.277	8.702 ***
H2: Social → USE	+	0.248	9.201 ***
H3: Informational → USE	+	0.442	14.047 ***

* Ten rel. = Tension release/entertainment motivation. *** *p* < 0.01.

**Table 5:** Results of invariance measurement testing using permutation (self-perception of level as a player).

Const.	Conf. Inv.	Compositional Invariance (Correlation = 1)		Equal Mean Assessment			Equal Variance Assessment			Measurement Invariance Established
		C = 1	Confidence Interval	Differences	Confidence Interval	Equal	Differences	Confidence Interval	Eq.	
INF *	Yes	1	[0.999, 1]	−0.033	[−0.125, 0.118]	Yes	0.153	[−0153, 0.134]	No	Partial
TEN/REL *	Yes	0.998	[0.998, 1]	0.036	[−0.136, 0.120]	Yes	0.162	[−0.126, 0.125]	No	Partial
SOC *	Yes	0.997	[0.994, 1]	0.286	[−0.121, 0.127]	No	0.482	[−0.258, 0.279]	No	Partial
USE *	Yes	0.999	[0.999, 1]	−0.064	[−0.129, 0.128]	Yes	−0.086	[−0.194, 0.182]	Yes	Total

* INF: informational motivation; TEN/REL: Tension release/entertainment motivation; SOC: Social motivation.

**Table 6:** Multi-group analysis by self-perception of level as a player.

Relationships	Path Coefficients		Path Coefficient Difference	Henseler’s MGA	Welch–Satterthwait Test	
	Basic Player β1	Advanced Player β2	β1-β2	*p*-Value	Student-*t*	*p*-Value
INF * → USE	0.476 **	0.339 **	0.137	0.030 *	2.160	0.031 *
TEN/REL * → USE	0.230 **	0.338 **	−0.108	0.081	1.753	0.080
SOC * → USE	0.238 **	0.294 **	−0.056	0.350	0.933	0.351

* INF: informational motivation; TEN/REL: Tension release/entertainment motivation; SOC: Social motivation. * *p* <0.05, ** *p* <0.01.
